# A Novel High Content Angiogenesis Assay Reveals That Lacidipine, L-Type Calcium Channel Blocker, Induces In Vitro Vascular Lumen Expansion

**DOI:** 10.3390/ijms23094891

**Published:** 2022-04-28

**Authors:** Dorota A. Nawrot, Lutfiye Yildiz Ozer, Ayman Al Haj Zen

**Affiliations:** 1 BHF Centre of Research Excellence, Division of Cardiovascular Medicine, Radcliffe Department of Medicine, University of Oxford, Oxford OX3 9DU, UK; dorota.nawrot@cmd.ox.ac.uk; 2 Alzheimer’s Research UK, Oxford Drug Discovery Institute, Target Discovery Institute, Nuffield Department of Medicine, University of Oxford, Oxford OX3 7FZ, UK; 3 College of Health and Life Sciences, Hamad Bin Khalifa University, Education City, Doha P.O. Box 34110, Qatar; lozer@hbku.edu.qa

**Keywords:** angiogenesis, in vitro angiogenesis assay, high-content screening, lumen formation, lacidipine

## Abstract

Angiogenesis is a critical cellular process toward establishing a functional circulatory system capable of delivering oxygen and nutrients to the tissue in demand. In vitro angiogenesis assays represent an important tool for elucidating the biology of blood vessel formation and for drug discovery applications. Herein, we developed a novel, high content 2D angiogenesis assay that captures endothelial morphogenesis’s cellular processes, including lumen formation. In this assay, endothelial cells form luminized vascular-like structures in 48 h. The assay was validated for its specificity and performance. Using the optimized assay, we conducted a phenotypic screen of a library containing 150 FDA-approved cardiovascular drugs to identify modulators of lumen formation. The screening resulted in several L-type calcium channel blockers being able to expand the lumen space compared to controls. Among these blockers, Lacidipine was selected for follow-up studies. We found that the endothelial cells treated with Lacidipine showed enhanced activity of caspase-3 in the luminal space. Pharmacological inhibition of caspase activity abolished the Lacidipine-enhancing effect on lumen formation, suggesting the involvement of apoptosis. Using a Ca^2+^ biosensor, we found that Lacipidine reduces the intracellular Ca^2+^ oscillations amplitude in the endothelial cells at the early stage, whereas Lacidipine blocks these Ca^2+^ oscillations completely at the late stage. The inhibition of MLCK exhibits a phenotype of lumen expansion similar to that of Lacidipine. In conclusion, this study describes a novel high-throughput phenotypic assay to study angiogenesis. Our findings suggest that calcium signalling plays an essential role during lumen morphogenesis. L-type Ca^2+^ channel blockers could be used for more efficient angiogenesis-mediated therapies.

## 1. Introduction

The formation of a new microvascular network, or angiogenesis, is a vital process for development, tissue homeostasis, repair and regeneration throughout life in both health and disease [1,2]. Sprouting angiogenesis consists of timely coordinated multiple cellular steps, starting with the directed endothelial cell sprouting, growth, and branching, and finalizing with lumen formation and maturation [3,4]. Aberrant regulation of any step would result in establishing an unfunctional vascular network that contributes to various disorders such as cancer, ischemic vascular disease, neurodegeneration, and immune diseases [5,6]. In vitro angiogenesis assays have been instrumental tools that allowed the investigation of blood vessel formation mechanisms and helped develop potential angiogenic-based therapeutics [7,8,9]. In these cellular assays, endothelial network formation is triggered by pro-angiogenic growth factors. Additionally, extracellular matrix components are usually integrated into the assay setting since the ECM provides the physical microenvironmental cues through which endothelial cells navigate, polarize, and self-organize into tubular branched structures [10,11]. However, most of these cell-based assays depict part of the process, intending to extrapolate and understand the whole process of blood vessel formation [12,13].

Over the past decades, the endothelial tube formation assay has been used widely to assess the angiogenic capacity of endothelial cells due to it being easy to perform [14]. It is quick, quantitative, and incorporates many steps of angiogenesis: adhesion, cell motility, branching, and tube formation [15,16]. Nevertheless, tube formation has several disadvantages; the formation of endothelial tubes on Matrigel is so quick that specific steps of angiogenesis might be skipped, such as cell invasion, proliferation, and lumen formation [10,17]. In addition, the assay uses Matrigel, a potent differentiation inducer for many other cell types; the stimulatory effects of Matrigel on the endothelial cell morphogenesis could mask the specific effects of angiogenic regulators [18]. In contrast, other in vitro models where endothelial cells are embedded in ECM gel include type I collagen. Endothelial cells can invade the ECM and form stable lumen-containing tubules preserved for several days [19,20]. However, these assays are usually unwieldy to use for high-throughput screening settings.

This study combined the two angiogenesis assay settings that use type I collagen gel and Matrigel to develop a more comprehensive two-dimensional (2D) phenotypic assay to simultaneously capture the critical steps of blood vessel morphogenesis, such as tubulogenesis, branching, cell polarization, and lumen formation. Furthermore, we conducted a high-content screen of a focused drug library using our optimized assay. We have identified that the attenuation of calcium signalling induces in vitro vascular lumen formation through apoptosis induction.

## 2. Results

### 2.1. Optimization of High-Content Imaging 2D Angiogenesis Assay

**Figure 1:**
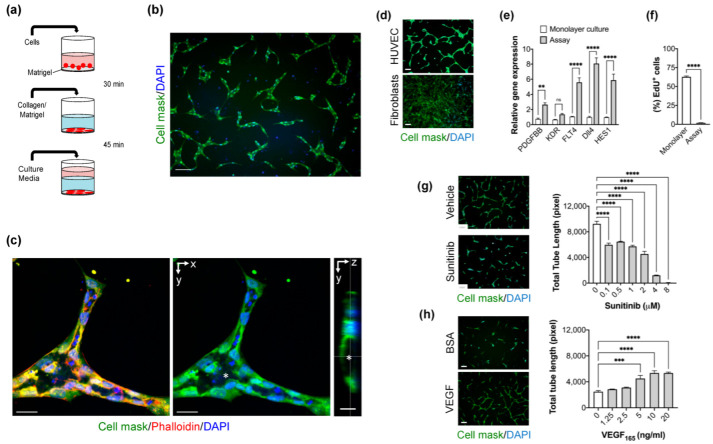
**Optimization and validation of the novel 2D angiogenesis assay.** (**a**) Workflow of the assay: HUVECs were seeded on coated 96-well plates with a thin layer of Matrigel. Extracellular matrix gel (type I collagen/Matrigel) was added on adhered HUVEC. Following gel polymerization, media was added and incubated for 48 h. (**b**) Representative fluorescent image showing the vascular-like structures formed by HUVEC cultured with type I collagen/Matrigel mixture. Cells were stained with HCS Cell Mask (green), and DAPI (Blue). Scale bar = 100 μm. (**c**) Confocal Z-stack imaging of a vascular-like unit showing the lumen (*) formation is associated with polarized endothelial cells. Cells are stained with HCS Cell Mask (green), Phalloidin (Red), and DAPI (Blue). xy plane (middle) and yz plane (right). Scale bar = 30 μm. (**d**) Morphology of cultured fibroblasts compared to HUVEC using the angiogenesis assay. Scale bar = 100 μm (**e**) Levels of pro-angiogenic genes: *PDGFB*, *KDR*, *FLT4*, *Dll4*, and *HES1* mRNA expression detected by quantitative RT-PCR in HUVEC cultured in the angiogenesis assay compared to HUVEC cultured in monolayer settings. Error bars, mean ± SEM, *n* = 3. Unpaired *t*-test, ** *p* < 0.01, *** *p* < 0.001 compared with monolayer culture conditions. (ns) non significant. (**f**) The proliferative activity of HUVEC cultured in angiogenesis assay for 48 h as measured by EdU staining, *n* = 6 per condition, unpaired *t*-test, Data are mean ± S.E.M, ** *p* < 0.01, *** *p* < 0.001. (**g**) *Right panel*, the effect of the anti-angiogenic compound, Sunitinib, with various concentrations on the tube formation (total tube length) using the angiogenesis assay. Data are shown as mean ± S.E.M. *n* = 3 wells per concentration. one-way ANOVA followed by Bonferroni’s post hoc test, **** *p* < 0.0001 compared to the vehicle. *Left panel*, representative fluorescent image of the cultures treated with Sunitinib (4 μM) and DMSO (vehicle). Cells were stained with Cell Mask (green) and DAPI (blue). Scale bar = 100 μm. (**h**) Effect of VEGF on tube formation. The assay was conducted in growth factor-free media (EBM). *n* = 3 wells per concentration. Error bars, mean ± S.E.M, one-way ANOVA followed by Bonferroni’s post hoc test, **** *p* < 0.0001 compared to vehicle. *Left panel*, representative fluorescent image of the cultures treated with VEGF (20 ng/mL) and Bovine Serum Albumin “BSA” (control). Cells were stained with Cell Mask (green) and DAPI (blue). Scale bar = 100 µm.

To delineate the extracellular matrix (ECM) composition in our assay setting, we tested the effect of adding type I collagen gel, Matrigel, or type I collagen gel/Matrigel mixture on freshly seeded endothelial cells. In all cases, endothelial cells formed a tube-like network with different morphological features over 48 h (Figure S1). However, the endothelial cells cultured with an ECM mixture of type I collagen gel and Matrigel exhibited an appearance of well-defined vascular-like structures with signs of cell polarization (Figure 1b). Next, we analyzed confocal images of labelled newly formed endothelial tubes to assess the lumen formation. The endothelial cells were polarized to form a lumen. Apoptotic bodies were observed in the lumen centre, showing that the lumen was formed by cavitation. Tip/stalk cell differentiation was also detected in the vascular-like structures (Figure 1c). Live cell imaging reveals that seeded endothelial cells were elongated and displayed extensive migratory activity in the first hours. The endothelial cells begin rapidly to integrate with neighbouring cells generating vascular-like units. Less than 5% of the endothelial cells do not integrate into tubes (Movie S1). Matrigel has previously been reported to stimulate tube-like structures by several non-endothelial cell types such as fibroblasts [18]. Therefore, we examined whether dermal fibroblasts form tubular structures and found that dermal fibroblasts do not form any tubular structure (Figure 1d). This result confirms the assay specificity for endothelial cell differentiation and morphogenesis. To further characterize the angiogenic response of the assay, we evaluated the gene expression of five pro-angiogenic genes: *PDGFB*, *Dll4*, *KDR*, *FLT4*, and *HES1*, that are involved in the sprouting angiogenesis. We found that their expression is increased >2-fold or more compared to those in monolayer cultures in the first 24 h. However, the increase in *KDR* expression was not significant (Figure 1e). After 48 h of culture, endothelial cell proliferation was reduced as measured by EdU labelling, indicating the stability of newly formed tubes (Figure 1f). Next, we tested the assay performance by evaluating the effect of anti-angiogenic and pro-angiogenic agents. Total tube length was used as a measurement parameter for tube formation. We found that Sunitinib, a tyrosine kinase inhibitor of vascular growth factors, inhibits the formation of endothelial tubes in a dose-response manner (Figure 1g). The assay Z’ factor was measured as 0.72 at 2 μM and 0.76 at 4 μM. In contrast, VEGF stimulates tube formation of HUVEC cultured in a medium without vascular growth factors (Figure 1h). The Z’ factor for this assay was 0.4 at a VEGF concentration of 20 ng/mL. Taken together, the optimized assay captures many features of angiogenesis, including lumen formation and is suitable for high-throughput screenings.

### 2.2. High-Content Screening of Focused Small Molecule Library

**Figure 2:**
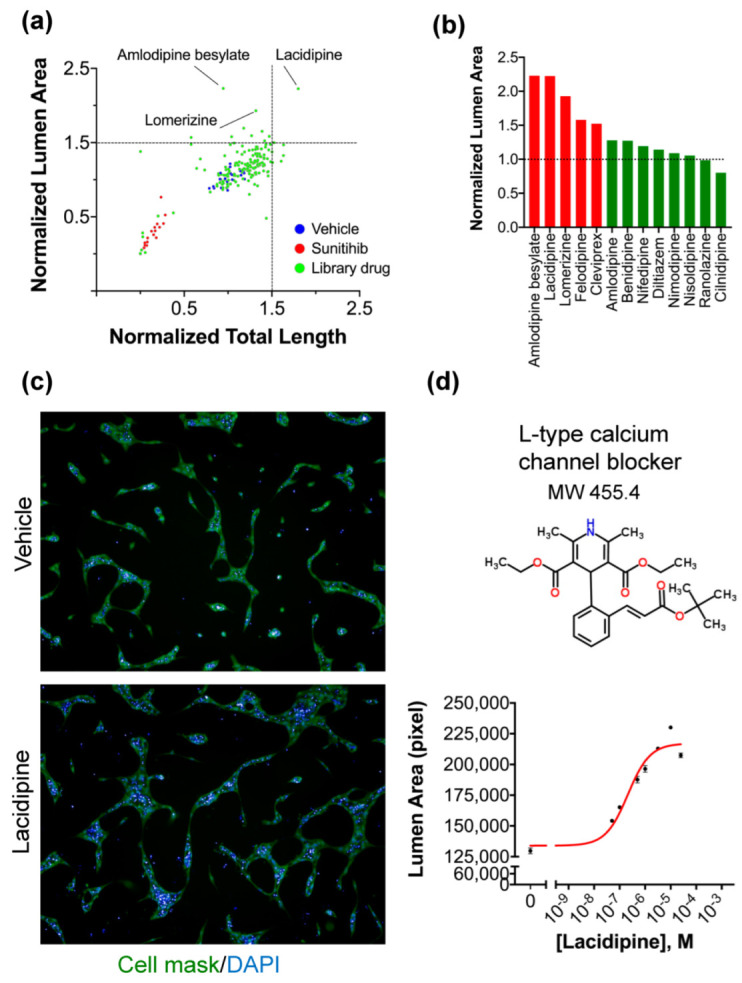
**High-content screening of the cardiovascular compound library using the angiogenesis assay and hit identification.** (**a**) Scatter-plot distribution showing the results of high-content screening. The lumen area and the total tube length were normalized to controls treated with vehicle (DMSO). Enhancer hits of the lumen area are indicated below and above the dotted lines (hit threshold was calculated as follows: median_(vehicle)_ + 4 × standard deviation_(vehicle)_). Negative controls (are shown in blue positive controls in red and drug library molecules in green. (**b**) Calcium blockers (in red) were identified as enhancers of lumen formation from the library. (**c**) Representative fluorescent images from negative control (vehicle) and Lacidipine (enhancer hit for lumen area). Scale bar = 100 µm. (**d**) Dose–response curve demonstrating the effect of Lacidipine on lumen size using the angiogenesis assay. Data are expressed as mean ± SEM. *n* = 3 per concentration. The EC_50_ was calculated as 250 nM.

To identify modulators of endothelial tube and lumen formation using the new optimized assay, we performed a high-content screening of a compound library containing 150 FDA-approved cardiovascular drugs in a 96-well plate format at a final concentration of 5 μM in duplicate. Sunitinib was used as a positive control. Using the image analysis approach, we quantified two morphological parameters: total tube length and lumen area. Treated wells were normalized to negative control (vehicle, DMSO) wells. Compounds that changed the total tube length or lumen area by >4 × SD from the mean of the DMSO-treated control wells were considered enhancer hits (Figure 2a). Of the 150 compounds, we found that several L-type calcium channel blockers with different chemical structures could enhance the formation of lumen compared to controls (Figure 2b). Lacidipine was selected for further validation and mechanistic studies. Dose-dependent effects of Lacidipine on the lumen area confirmed the screen results and demonstrated that the half-maximal effective concentration (EC_50_) of Lacidipine is (250 nmol/l) in this assay (Figure 2c). Our data shows that L-type calcium blockers enhance the endothelial lumen formation in our assay.

### 2.3. Effect of Lacidipine on Endothelial Cell Functions

**Figure 3:**
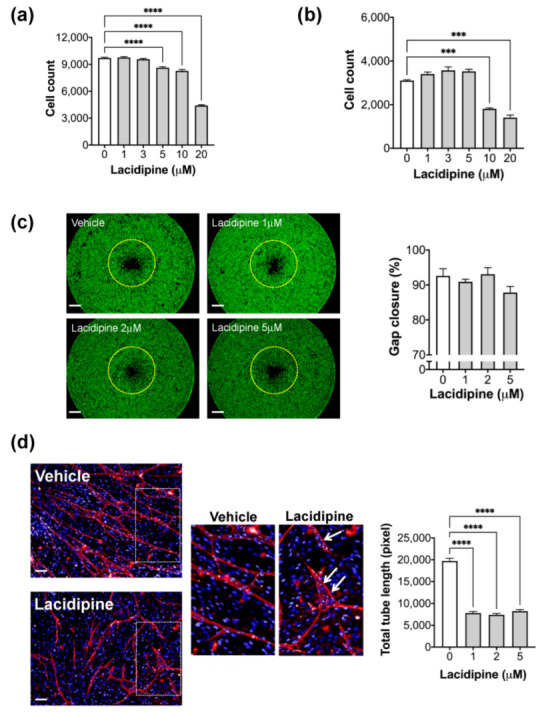
**Effect of Lacidipine on the endothelial cell functions.** Effect of Lacidipine on cell proliferation in HUVEC cultured as a monolayer (**a**) or in the angiogenesis assay (**b**); *n* = 6 per concentration, cell count was assessed after 48 h of assay initiation. Data expressed as mean ± SEM, one-way ANOVA followed by Bonferroni’s post hoc test, *** *p* < 0.001 compared with vehicle. (**c**) Representative fluorescence images following Oris^TM^ migration assay of HUVEC treated with vehicle or Lacidipine (1, 2, and 5 μM) for 22 h. The yellow dotted line represents the cell edge at 0 h after removing the stoppers. Scale bar = 0.5 mm. (**Right panel**): quantification of cell migration. The graph represents the average per cent of gap closure ± S.E.M. from six independent experiments. Unpaired student’s *t*-test was used. No significant differences between groups were detected. (**d**) The effect of Lacipidine (1, 2, and 5 μM) on tube formation was assessed in a coculture angiogenesis assay where HUVECs were plated on a confluent human dermal fibroblast layer. The medium containing the compound was refreshed on days 3 and 5 following the plating of endothelial cells. Cocultures were stained with an antibody against CD31 and imaged 7 days after endothelial cell plating. The representative fluorescence images of the cocultures treated with Lacidipine (5 μM) and DMSO (vehicle). *Inset*, “White arrows” indicate the newly formed lumens. (**Left panel**), total tube length was quantified in nine fields for each well (*n* = 6 wells per group). Error bars, mean ± SEM, one-way ANOVA followed by Bonferroni’s post hoc test, **** *p* < 0.001 as compared with vehicle. Scale bar = 100 μm.

Intracellular calcium signalling is a ubiquitous secondary messenger in many signal transduction pathways that activate cellular responses such as cell proliferation and migration [21,22]. Therefore, we wondered whether Lacipidine affects endothelial cell growth and migration as both processes are involved in angiogenesis. In monolayer cell culture, Lacidipine reduced cell growth by <10% at a 5 μM concentration over 48 h (Figure 3a). In contrast, Lacidipine did not affect the growth of endothelial cells when they were cultured in the angiogenesis assay at a similar concentration (Figure 3b). At a higher concentration (>10 μM), we found that Lacipidipine reduced cell growth by >50%, suggesting a cytotoxic effect of a high dose. Using Oris^TM^ cell migration assay, endothelial cell migration was not significantly affected upon Lacidipine treatment for 21 h (Figure 3c). In contrast, Lacipidine reduced the total tube length by >50%, even at a lower concentration (1 μM), when assessed by endothelial cells co-cultured with fibroblast for five days (Figure 3d). This suggests that long-term exposure to Lacipidine, the L-type calcium channel blocker, restricts the endothelial tube formation capacity and induces an early formation of the lumen, which is a sign of vascular stabilization and maturation.

### 2.4. Lacidipine Enhances Lumen Formation by Inducing Apoptosis

**Figure 4:**
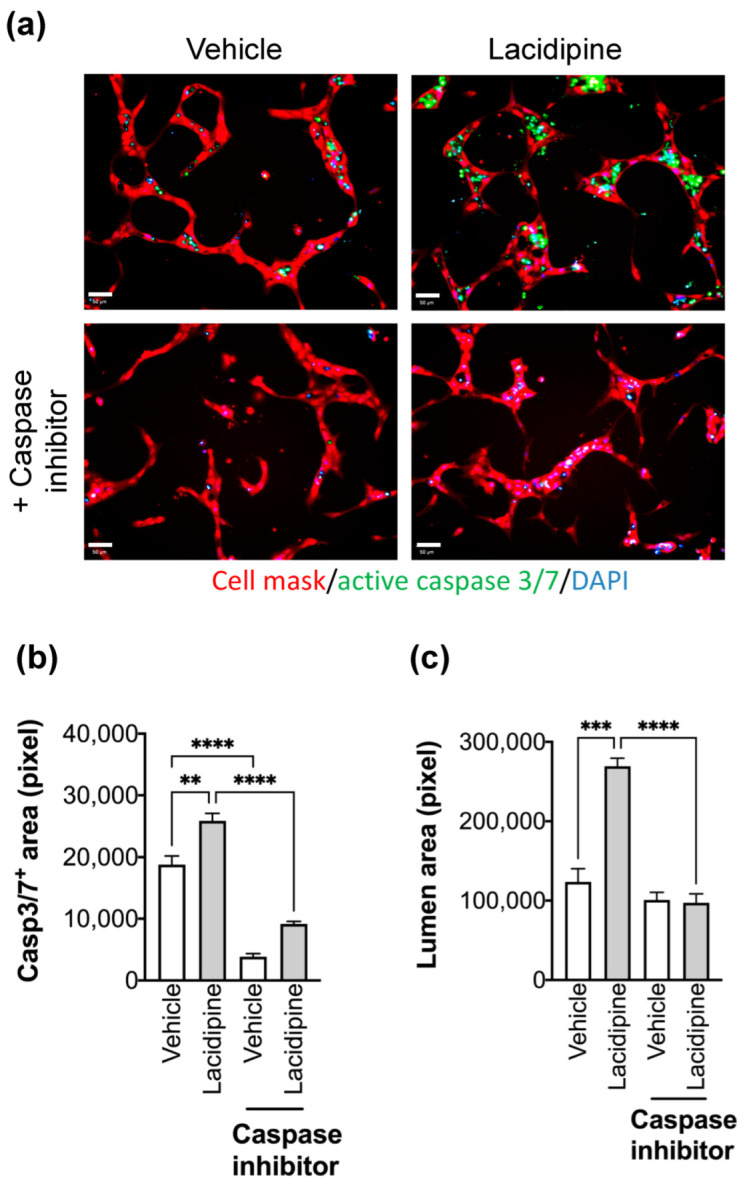
**Effect of Lacidipine on the lumen formation is mediated by endothelial cell apoptosis.** (**a**) Cell apoptosis was detected by assessing the caspase 3/7 activity (CellEvent™ Caspase-3/7 Green Detection Reagent, Invitrogen) in the presence of Lacipidine (5 μM) or vehicle (DMSO). The caspase inhibitor was Z-VAD-FMK (100 μM). Cells were fixed after labelling the active caspase 3/7 (green), nuclei were counterstained with DAPI (blue), and the cell body was stained with Cell Mask deep red (red). Scale bar = 50 μm. Caspase 3/7 positive area (**b**) and lumen area (**c**) were quantified in nine fields for each well (*n* = 4 per condition), data are expressed as mean ± S.E.M, one-way ANOVA followed by Bonferroni’s post hoc test, ** *p* < 0.01, *** *p* < 0.001, **** *p* < 0.0001.

In similar flow-independent models of epithelial acini, apoptosis has been suggested to play an essential role in creating a lumen space [23]. To assess whether apoptosis enhances lumen formation by Lacidipine, we measured the caspase-3 activity since it is an indispensable effector for apoptotic commitment. In our assay, we detected cells in the luminal space of vascular-like structures stained positively for activated caspase-3, and these cells exhibited fragmented nuclei upon staining with the nuclear dye, DAPI. Lacidipine increases the number of apoptotic cells detected in the luminal space. The blockade of caspase activity abolished the Lacidipine-enhancing effect on lumen formation (Figure 4).

### 2.5. Effect of Lacidipine on the Intracellular Ca^2+^ Dynamics in Endothelial Cells during Endothelial Lumen Formation

**Figure 5:**
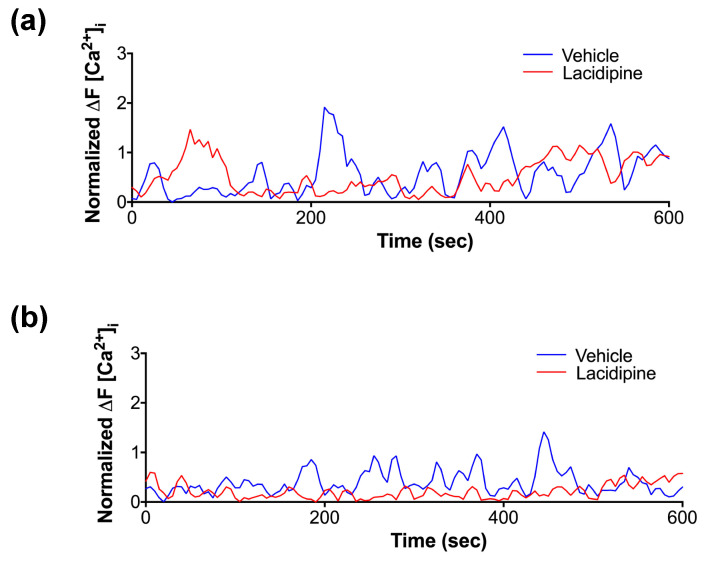
**Ca^2+^ imaging to detect the endogenous intracellular changes of Ca^2+^ at early and late stages of tube formation.** To monitor the intracellular temporal Ca^2+^ dynamics, HUVEC were stably infected with genetically encoded Ca^2+^ FRET sensor (GCaMP) using a lentiviral vector. Cells were time-lapse imaged using the high-content imaging system (Operetta) with 20× objective at 5 s intervals for 10 min. The fluorescent signal was quantified using Harmony imaging software. For each individually tracked cell, a spherical region of interest to avoid overlapping with adjacent cells. The mean fluorescent intensity of all cells at every time point was normalized to the baseline fluorescent intensity of GCaMP6. The graph traces are representative of three independent experiments. A total of 25–40 cells were included in the quantification for each experiment. (**a**) Representative traces illustrating a pattern of repeated _i_Ca^2+^ spikes in HUVEC at an early stage (1 h) of the angiogenesis assay. (**b**) Representative traces showing that the generated _i_Ca^2+^ spikes in the presence of Lacidipine (5 μM) for 24 h.

**Figure 6:**
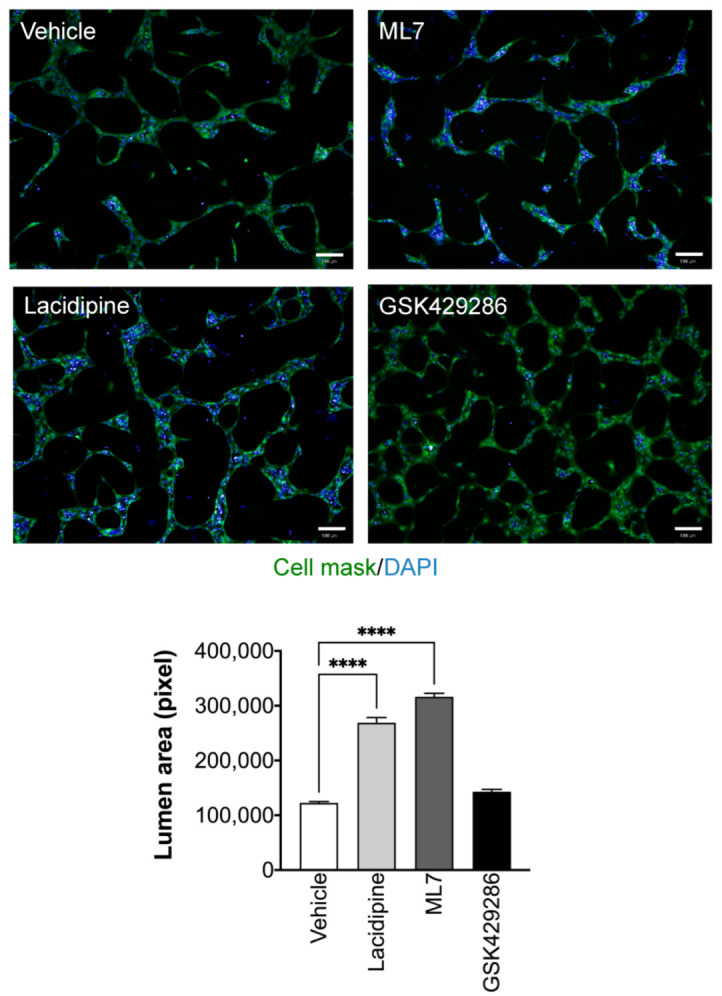
**Lacidipine induces lumen formation through MLCK inhibition rather than ROCK inhibition.** Representative fluorescence images of ML7, a myosin light chain kinase inhibitor (1 μM), which enhances the lumen formation with a similar phenotype of Lacidipine. However, the ROCK inhibitor (GSK429286, 5 μM) did not enhance the lumen formation. *n* = 4 per condition, data are expressed as mean ± S.E.M, one-way ANOVA followed by Bonferroni’s post hoc test, **** *p* < 0.0001 as compared to vehicle; scale bar = 100 μm.

It has been shown that tip migratory endothelial cells exhibited oscillatory activity of intracellular Ca^2+^ during sprouting angiogenesis [24]. Therefore, we evaluated the intracellular Ca^2+^ dynamics in the endothelial cells during tube formation in our assay. We used the Ca^2+^ imaging method to precisely detect the endogenous intracellular changes of Ca^2+^ at the early and late stages of tube formation. During the first hour, endothelial cells exhibited a pattern of repeated Ca^2+^ spikes. The addition of Lacidipine only reduced the intensity of generated Ca^2+^ spikes (Figure 5a). In contrast, after 24 h, the Ca^2+^ intensity of spikes was lower compared to those detected in the first hour and the spikes were observed more often in the endothelial tip cells (Figure 5b; Movie S2). Lacidipine damps further reduced endothelial Ca^2+^ oscillations during the lumen formation (Figure 5b; Movie S3). It has been demonstrated that Ca^2+^ oscillations (repeated spikes) in response to VEGF that stimulate the migratory behaviour in endothelial cells involve the activation of myosin light chain kinase (MLCK), a Ca^2+^-sensitive kinase [25]. Therefore, we investigated whether MLCK mediates the process of lumen formation in the current model. We found that the pharmacological blocking of MLCK activity enhances the lumen formation with signs of apoptosis in the intra-luminal space, producing a similar phenotype of Lacidipine. However, the inhibition of ROCK activity did not enhance the lumen formation (Figure 6), also known as an effector to phosphorylate MLC. Based on these results, we conclude that inhibition of calcium influx reduces endothelial Ca^2+^ oscillations, which are associated with the increase of lumen formation mediated by apoptosis, and this effect could be MLCK-dependent.

### 2.6. Discussion

In this study, we optimized the settings of a new phenotypic 2D-cell-based assay for evaluating endothelial morphogenesis and drug discovery screens targeting the formation of new blood vessels. Endothelial cells were cultured beneath a mixture of type I collagen and Matrigel, giving rise to vascular-like structures for two days. Compared to the conventional Matrigel tube formation assay, these structures were stable for a longer period and their formation was specific to the primary endothelial cell type. The newly formed vascular-like structures recapitulate the main hallmarks of an interconnected capillary network in vivo, including the formation of growth-arrested polarized endothelium with a lumen. The optimized assay is straightforward, reproducible, and easy to quantify. Although the assay reflects the complexity of the vessel formation process, the image acquisition and analysis are performed under two-dimensional settings, making the assay suitable for conducting primary high-throughput image-based drug screens at a large scale. Maintaining the balance between endothelial cell growth and migration during angiogenesis is essential to forming a functional vascular network in vivo [26]. In our assay, endothelial cells in the newly formed tubes showed a low proliferating status as assessed by DNA synthesis level despite mitogenic growth factors. The low proliferating activity was associated with an overexpression of specific tip and stalk cell genes such as *PDGFB*, *FLT4*, *DLL4*, and *HES1* [27,28]. We detected significant migratory activity of endothelial cells during their assembly and tube formation using live imaging. In contrast, when endothelial cells are seeded on the Matrigel, the formation of endothelial tubes is dependent on the extracellular matrix degradation [29,30] and on cellular forces exerted on the substrate, with a minimal role in the cell migration. We also showed that our new model could assess the angiogenic potential of compound candidates by measuring the parameter of tube length with a high value of Z’ factor suitable for high-throughput screening.

One important aspect of this assay is the formation of the lumen by cavitation. In the current model, we confirmed the involvement of apoptosis in lumen formation by detecting the increase of caspase-3/7 activity exclusively in the luminal space. The role of apoptosis has been suggested as the underlying mechanism of glands morphogenesis and lumen formation [31]. In contrast, different cellular mechanisms have been described for the vascular lumen, such as cell and cord hollowing [32]. Cell hollowing is formed by intracellular fusion of endothelial vacuoles, which are resulted from pinocytosis. This process is followed by cord hollowing in which the intracellular vacuoles merge with those of neighbouring endothelial cells to form a continuous luminal space in the developing blood vessels [33]. For larger vessels, the lumen is generated when an extracellular gap is formed between neighbouring endothelial cells [34]. More recently, it has been shown that lumen expansion coincides with the formation of unidirectional spherical bleb-like protrusions in the new sprouts. Indeed, these bleb-like structures are similar to those usually observed in apoptosis, but they have inverted polarity (termed inversed membrane blebs). The process of inverse membrane blebbing is driven by blood flow and requires actomyosin activity [35].

Earlier studies reported the presence of products of necrosis or apoptotic bodies in the luminal space of endothelial tubulogenesis models in vitro [36,37,38,39]. Furthermore, in the core stroma of villi, apoptotic cells were detected at the centre of the capillary lumen during the very early stages of placental vasculogenesis and angiogenesis [40]. It has been suggested that the apoptosis of differentiating cells in the primitive vascular islands is responsible for the early lumen formation before the blood flow initiation. This process could also be relevant in vivo for the vascular development of blood islands of the vertebrate yolk sac [41]. However, further studies are required to investigate the potential role of apoptotic signalling pathways in the lumen formation in early embryogenesis in the absence of blood flow before the heart starts beating. Altogether, the current assay offers insights into many aspects of endothelial morphogenesis and the development of vascular systems in vivo. Despite the assay representing a two-dimensional (planar) model of vascular growth, it can simulate sprouting angiogenesis because endothelial cells are embedded in three-dimensional extracellular matrix gel.

This study also represents a high-content drug discovery screen to identify candidate compounds that regulate the endothelial lumen formation in a focused library consisting of 150 FDA-approved compounds for cardiovascular diseases. We have initially identified L-type calcium channel blockers (Amlodipine besylate, Lacidipine, Lomerizine, Felodipine, Cleviprex) as enhancers of the lumen area from the screen. Some calcium blockers from the library, such as Nifedipine and Diltiazem, did not produce a strong phenotype due to their chemical instability or not reaching the optimal effective dose [42] over a prolonged culture period. Lacidipine and the other identified calcium channel blockers are voltage-dependent L-type calcium (Cav1.2) channel blockers [43]. They are commonly used to treat primary hypertension for their effectiveness to vasodilate arterioles through their actions in reducing the contractile function of smooth muscle cells. Because endothelial cells are non-excitable cells, it has been supposed that they are not dependent on voltage changes for alterations in intracellular Ca^2+^ as smooth muscle cells. Nevertheless, many studies provided evidence that endothelial cells expressed all subunits of voltage-gated Ca^2+^ channels, and these channels are functional [44]. Intracellular Ca^2+^ concentration is mainly maintained by increased Ca^2+^ influx through the plasma membrane Ca^2+^ channels or Ca^2+^ release from cellular storage such as the endoplasmic reticulum [45]. In endothelial cells, the acute exposure to vascular growth factors and vasoactive molecules triggers an initial spike which reflects PI(1,4,5)P_3_-induced release of intracellular Ca^2+^ storage, followed by a sustained component of the intracellular calcium response due to Ca^2+^ influx across the plasma membrane from the extracellular space [46]. At low-dose agonist stimulation, the Ca^2+^ signal may adopt an oscillatory pattern driven by the balance between intracellular Ca^2+^ release and Ca^2+^ influx from the extracellular space, correlated with their proliferative or migratory activity. In vascular endothelium, it has been reported that the depletion of intracellular Ca^2+^ stores can activate the calcium influx through agonist Ca^2+^-selective channels [47,48,49,50]. This calcium influx is usually required in the case of long-term cellular responses [51]. Yet, the long-term effect of the L-type calcium channel on endothelial cell functions is poorly investigated during endothelial morphogenesis. In our study, Lacidipine did not affect the endothelial cell migration in the short term, whereas endothelial tube formation is reduced in the long term. These phenotypic findings are in line with the Ca^2+^ dynamics results. In our study, in the short term, the endothelial Ca^2+^ oscillations were slightly reduced after L-type calcium channel blocking by Lacipidine. However, these oscillations were blocked at long-term incubation with Lacidipine. Taken together, our data suggest that calcium influx through L-type calcium channels is an essential route for refilling the intracellular calcium stores in long-term responses such as tube formation. Blocking the Ca^2+^ oscillations leads to a halt in the angiogenic switch in endothelial cells and enhances the process of lumen formation.

Intracellular calcium concentration is an essential regulator of MLCK activity. Upon increasing intracellular calcium level, calcium binds to calmodulin which activates MLCK. MLCK phosphorylates regulatory myosin light chains (MLC) at two residues, serine 19 and threonine 18 [52]. The MLC phosphorylation increases the myosin ATPase activity, activating actomyosin contractility and loosening endothelial cell–cell adhesion [53]. MLCK-mediated MLC phosphorylation and actomyosin cytoskeleton is involved in many physiological responses such as apoptotic blebbing [54], cell migration [55], endocytosis [56], and endothelial barrier function [57]. Our findings showed that MLCK inhibition reproduced a similar lumen expansion phenotype when an L-type calcium channel blocker inhibits calcium influx. Moreover, lumen expansion was associated with an increase of apoptotic bodies in the luminal space. Previous studies showed that inhibiting MLCK activity pharmacologically or blocking antibodies induces apoptosis in vitro and in vivo [58]. The increase of MLCK activity is an upstream event of caspase activation and precedes apoptosis. In addition to MLCK, ROCK increases phosphorylation of MLC by inhibiting MLC phosphatase [59] or by direct phosphorylation of MLC [60]. The ROCK inhibitor did not enhance the lumen expansion as calcium channel blockers, suggesting that the primary input into MLC dephosphorylation is via MLCK rather than ROCK. Pharmacologic ROCK inhibition has been reported to reduce pp-RLC but not p-RLC levels in epithelial cells and thrombin-activated porcine aortic endothelial cells [61]. In contrast, pharmacologic MLCK inhibition (via ML-9 or Ca^2+^ depletion) has been observed to affect neither phosphorylation state significantly [62,63].

In summary, we present that our current angiogenesis assay could be used for performing high-throughput screens of drug libraries. Furthermore, our findings highlight an important role of L-type calcium channels in regulating the endothelial Ca^2+^ oscillations during angiogenesis. Our observations are required for further in vivo studies to investigate the link between calcium signalling and apoptotic-related pathways in lumen formation during embryogenesis and postnatal life. Angiogenesis relies on forming an opening lumen to allow perfusion to the tissue in demand. Forming effective and patent microvasculature is a fundamental goal of vascular normalization therapy. For instance, blood flow at the tumour site becomes inefficient in cancer due to the abnormal and leaky vasculature associated with the tumour microenvironment. As such, the identification of therapeutic compounds that effectively enhance vascular stability and lumen formation can be beneficial in reducing tumour metastasis and improving the tissue delivery of anti-tumoural drugs. It will be important to focus further pre-clinical works on the potential of L-type calcium channels as angiogenesis targets combined with anti-cancer drugs.

## 3. Materials and Methods

### 3.1. Cell Culture

Pooled primary Human Umbilical Vein Endothelial Cells (HUVEC) were purchased from Lonza. Cells were maintained in an endothelial cell growth medium (EGM-2, Lonza). Cells between passages 2 and 8 are used for the experiments. Adult human dermal fibroblasts (HDF-Ad) were purchased from LONZA and maintained in human fibroblast expansion basal medium media (ThermoFisher Scientific, Basingstoke, UK) supplemented with a low serum growth supplement (ThermoFisher Scientific, Basingstoke, UK).

### 3.2. Chemical Compounds

We constructed a subset library of approximately 150 compounds with research area annotation of cardiovascular disease from the FDA-approved drug library (HY-L022, MedChemExpress). Compounds were dissolved in dimethylsulfoxide (DMSO), transferred to 96-well plates, and stored at −80 °C. A calcium channel blocker (Lacidipine), and selective MLCK inhibitor (ML7) were purchased from Sigma. Caspase inhibitor (Z-VAD1-FMK) and selective ROCK1/2 inhibitor (GSK429286) were purchased from TOCRIS.

### 3.3. Angiogenesis Assay

Microplates with a 96-well format were coated with Corning Matrigel Matrix at 250 μg/mL for 1 h at 37 °C. Excess liquid was removed, and HUVEC were seeded at a density of 15,000 cells per well. The cells were incubated for 30 min at 37 °C to allow HUVEC adherence. The culture media was replaced by an ECM gel mixture (50 μL) containing 30% Corning^®^ Collagen I Rat Tail, High-Concentration (8–9 mg/mL), 25% Corning Matrigel Matrix (10 mg/mL), 0.07% NaOH (1 N), and 45% growth medium (EGM-2). The plates were incubated for an additional 30 min at 37 °C, 5% CO_2_ to allow polymerization of extracellular matrix (ECM) gel. Pre-warmed fresh EGM-2 medium (100 μL) was added to the top of the ECM gel (Figure 1a). The cell plates were incubated for 48 h at 37 °C with 5% CO_2_ to allow the formation of vascular-like structures. At the experiment end, the plates were washed with phosphate-buffered saline (PBS) and fixed with 4% paraformaldehyde for 30 min at room temperature. Cells were permeabilized with 0.1% Triton X-100 for 15 min at room temperature. The plates were incubated with a mixture solution containing Alexa Fluor^®^ 568 Phalloidin, 1:100 (Thermo Fisher Scientific, Basingstoke, UK), HCS Cell Mask green stain, 1:5000 (Thermo Fisher Scientific, Basingstoke, UK), and Hoechst 33,342 1:500 (Thermo Fisher Scientific, Basingstoke, UK) for 45 min at room temperature. The plates were washed three times with a washing buffer (PBS/0.05% Tween-20) for 15 min.

### 3.4. High-Content Imaging and Analysis

Following the staining procedure, plates were imaged with a high-content imaging system (Operetta, PerkinElmer, Rodgau, Germany) at 10× magnification, nine fields per well. Images were analyzed to extract the total tube length and branching point parameters using Metamorph (Molecular Devices, San Jose, CA, USA) image analysis software. We used an automated sequence in the IN Cell Developer Toolbox 1.9.2 software (GE) to quantify the lumen area of the vascular-like structures (Figure S2). To observe the dynamics of vascular-like structure formation, the time-lapse live imaging was performed using GFP lentiviral-transduced HUVEC in a heated and CO_2_-controlled and integrated chamber within the high-content imaging system as described previously [64]. Images were captured with 10× objective at 15 min intervals for a total of 16 h.

### 3.5. Endothelial Cell Proliferation Assay

DNA synthesis was assessed using Click-iT EdU Alexa Fluor 488 HCS kit (Thermo Fisher Scientific, Basingstoke, UK) according to the manufacturer’s protocol [64]. Briefly, EdU-labelling medium (10 μM final concentration) was added to the culture plate and incubated for 6 hr before fixation. Next, the culture plates were fixed with 4% paraformaldehyde and treated with 0.1% Triton X-100 for 15 min. After washing with PBS, the cells were stained with a Click-iT reaction cocktail working solution at room temperature for 30 min. The cells were stained with DAPI at room temperature for 20 min. The plate was imaged and quantified using a high-content imaging system (Operetta). The total number and percentage of EdU-positive cells were calculated from nine fields/well using a 10× objective (Harmony software, PerkinElmer, Rodgau, Germany).

### 3.6. Endothelial Migration Assay

HUVEC were seeded at 20,000 cells per well in 96-well plates fitted with stoppers (Oris™ Cell Migration Assay, Platypus Technologies). Cells were incubated overnight at 37  °C and 5% CO_2_ before removing the stoppers. Cells were incubated with Lacidipine (1, 2, and 5 μM) or DMSO (vehicle) for 22 h after removing the stoppers. Cells were fixed with PFA 4% and stained with HCS green Cell Mask (Thermo Fisher Scientific, Basingstoke, UK). The plates were imaged using the high-content imaging system (Operetta) (×2 magnification) at hour 0 (bright field) and hour 24 (after cell staining) upon removal of stoppers. The gap areas were quantified using Image J NIH software. The effect of Lacidipine on endothelial cell migration was assessed by calculating the gap closure (%) using the following formula:

$$Gap\;closure\;\left(\%\right)=\left(1-\frac{Gap\;area{\;}_{24\;h}}{Gap\;area{\;}_{0\;\;h}}\right)\times 100$$

### 3.7. Quantitative Reverse Transcriptase Polymerase Chain Reaction

Total RNA was isolated from cultured endothelial cells using the Qiagen RNeasy Micro Kit according to the manufacturer’s protocol. cDNA was prepared with QuantiTect Reverse Transcription Kit (Qiagen). SYBR green quantitative polymerase chain reaction was carried out with the following QuantiTect primer assays of angiogenic genes: Hs_FLT4_1_SG (QT00063637), Hs_KDR_1_SG (QT00069818), Hs_Dll4_1_SG (QT00081004), Hs_HES1_1_SG (QT00039648), and Hs_PDGFB_1_SG (QT00001260). The data were normalized to the endogenous control *HPRT1*, Hs_HPRT1_1_SG, (QT00059066). Fold changes were calculated using the comparative ddCT (threshold cycle) method (BioRad, Hercules, CA, USA).

### 3.8. Coculture Angiogenesis Assay

Adult human dermal fibroblasts were seeded at 15,000 cells per well. The next day, HUVEC were plated at 4000 cells per well on the confluent fibroblast monolayer in a 96-well plate as described previously [64]. The cocultures were supplemented with an EGM-2 medium. Endothelial tubes were stained using a mouse antihuman CD31 monoclonal antibody (ref. BBA7, R&D systems) at a 1:100 dilution and goat Alexa 568-conjugated anti-mouse IgG secondary antibody (Invitrogen) at a 1:400 dilution. Nuclei were visualized by Hoechst staining. The endothelial tubes were imaged automatically at 10× magnification using the high-content imaging system (Operetta). Nine fields were acquired from each well in each experiment. Quantification of the total tube length was performed automatically using Metamorph software version 7.7.7.0 (Molecular Devices).

### 3.9. Caspase 3/7 Activity Assay

We used CellEvent™ Caspase-3/7 Green Detection Reagent (ThermoFisher) to detect apoptotic cells. The detection reagent was added to live endothelial cells and incubated for 30 min. Cells were fixed and co-stained with HCS Cell Mask and DAPI. The apoptotic cells with activated caspase-3/7 exhibited bright green fluorescent signals in nuclei. The plates were imaged and quantified using high-content fluorescent microscopy (Operetta). The total cell number and percentage of activated caspase3/7-positive cells were calculated from 9 fields/well using a 10× objective (Harmony software, PerkinElmer).

### 3.10. Imaging Intracellular Calcium

To image intracellular calcium levels, HUVEC stably expressing GCaMP6, a genetically encoded calcium sensor, was obtained by transduction of GCaMP6-puro-lentiviral vector [65]. GCaMP is a fusion of Ca^2+^-binding protein calmodulin (CaM), M13 Ca^2+^/CaM-binding sequence from myosin light chain kinase (MLCK), and a circularly permuted green fluorescent protein. HUVECs expressing GCaMP6 were live imaged using high-content imaging with 20× objective after one hour and 24 h of treatment. The cells were imaged at 37 °C with 5% CO_2_ using the high-content imaging system (Operetta). Time-lapse images were collected every 5 seconds for 10 min. To quantify intracellular Ca^2+^ levels of individual endothelial cells at each time point, they were automatically tracked over time using Harmony software. ΔF was calculated as (F−F0)/F0, where F0 is the baseline fluorescent intensity of GCaMP6. Fluorescence changes in GCaMP6 of individual endothelial cells are represented as ΔF traces in the graphs, and the mean ΔF was calculated by taking the average of every ΔF/F0.

### 3.11. Statistical Analysis

Statistics were carried out using Prism GraphPad version 9. Two treatment groups were compared by unpaired *t*-test. Multiple group comparisons were analyzed by one-way or two-way analysis of variance with post hoc Bonferroni’s test. A *p*-value of < 0.05 was considered to be statistically significant. Several independent experiments were performed to guarantee the reproducibility of the findings. The Z’ factor was used to measure the performance of the assay [66].

## Data Availability

Not applicable.

## Supplementary Materials

The following supporting information can be downloaded at: https://www.mdpi.com/article/10.3390/ijms23094891/s1.
