# Supplementary material for: A Novel High Content Angiogenesis Assay Reveals That Lacidipine, L-Type Calcium Channel Blocker, Induces In Vitro Vascular Lumen Expansion
Source: Int J Mol Sci. 2022 Apr 28;23(9):4891. doi: 10.3390/ijms23094891 (PMC9100973; doi:10.3390/ijms23094891)
Supplement: Supplementary file 1 [file ijms-23-04891-s001.zip › Figure S1.pdf]

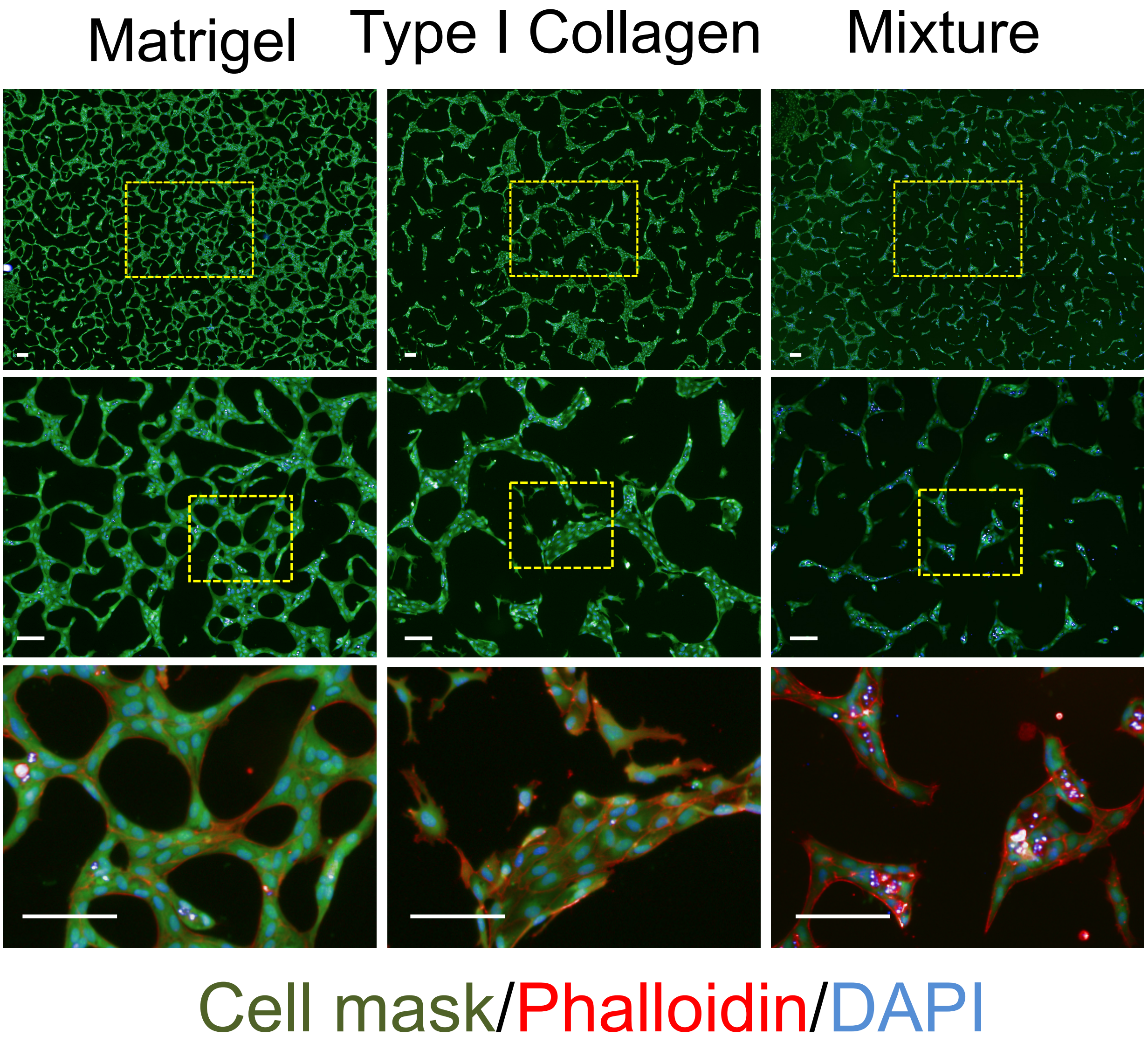

Figure S1. Effect of different ECM settings on endothelial morphogenesis. HUVECs were seeded on coated 96-well plates with thin layer of Matrigel. Matrigel, type I collagen gel, or Matrigel/type I collagen gel mixture was added. Cells were incubated for 48 hours. Next, the new forming structures were fixed and stained with Green Cell Mask (Cell body), Phalloidin (actin filaments), and DAPI (Nuclei). The fluorescent images of plates were acquired using high content imaging system (Operetta). Scale bar = 100 μm
