# Supplementary material for: A Novel High Content Angiogenesis Assay Reveals That Lacidipine, L-Type Calcium Channel Blocker, Induces In Vitro Vascular Lumen Expansion
Source: Int J Mol Sci. 2022 Apr 28;23(9):4891. doi: 10.3390/ijms23094891 (PMC9100973; doi:10.3390/ijms23094891)
Supplement: Supplementary file 1 [file ijms-23-04891-s001.zip › Figure S2.pdf]

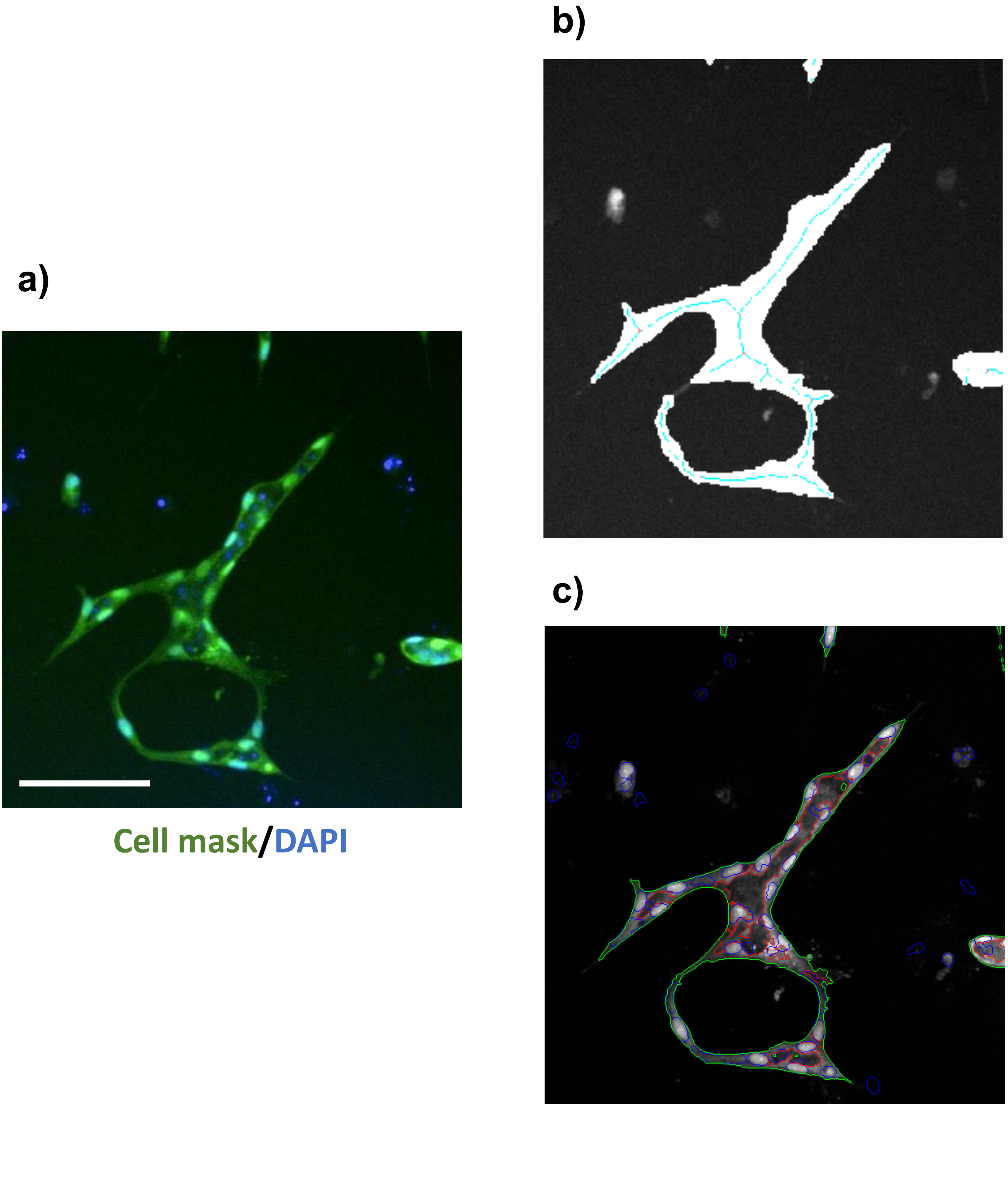

Figure 2S. Image analysis of the angiogenesis assay. a) the vascular-like structures were stained with cell mask (green) and DAPI (blue) to quantify the angiogenic features, scale bar=100 μM. **b)** an automated segmentation of vascular-like structures (white mask) to determine the tube skeleton (blue) and bansching points (red dot) using Metamorph image analysis software (Molecular Device). **c)** The protocol steps includes nuclei segmentation using the DAPI channel (blue line), the segmentation of vascular-like structures (green) using the green cell mask channel (green line), and lumen (cavity) segmentation with lower density signal of green cell mask stain (red line) within the structure region.
